# Discounting, Cognitive Inflexibility, and Antisocial Traits as Predictors of Adolescent Drug Involvement

**DOI:** 10.3389/fpsyg.2021.676250

**Published:** 2021-06-17

**Authors:** Laura Hernández, Diana Mejía, Laurent Avila-Chauvet

**Affiliations:** Psychology Department, Instituto Tecnológico de Sonora (ITSON), Ciudad Obregón, Mexico

**Keywords:** antisocial traits, discounting, cognitive flexibility, adolescents, drug use

## Abstract

Cognitive impairments, such as steep delay discounting, have been correlated with substance-related disorders. However, antisocial traits, cognitive inflexibility, and loss discounting have been barely considered despite having a high relationship with problematic consumption. This study aims to identify the predictive power of these variables in four types of drug use. Fifty-two adolescents (age range of 13 to 19 years) were assessed with a substance involvement test, four discounting tasks using $3,000, a card sorting test, and antisocial screening. Discriminant analysis with simultaneous estimation and varimax rotation was carried out. Function one included discounting of both losses, function two AT and CI, and function three probabilistic gains. The three functions explained 60.1% of the variance. The results show that preference for small and soon punishments and larger and unlikely punishments distinguished non-use and experimental use of moderate consumption and problematic consumption. High antisocial traits and low cognitive inflexibility distinguished experimental use groups of non-use. Risk-taking did not discriminate effectively between moderate consumption and problematic consumption. A replication of this study with a larger sample size is recommended to verify the results.

## Introduction

Substance-related disorder causes significant negative health consequences (loss of years of healthy life and death) and involves expensive healthcare and criminal justice costs (Gibbons, 2019). Several studies have focused on studying two types of risk factors for drug use. The first type is the preference for small immediate rewards (i.e., impulsive decision-making) instead of large, delayed rewards (i.e., self-control), which is also known as delay discounting (Green and Myerson, 2004). Steep delay discounting has been strongly associated with drug abuse because it is more pronounced in subjects with heavy use, poly-use, and substance-related disorder (SRD) (Green and Myerson, 2013; Janssen et al., 2015; Mejía Cruz et al., 2015; Mejía-Cruz et al., 2016; Moreira et al., 2015; Myerson et al., 2015; Moody et al., 2016; Quisenberry et al., 2016; Martínez-Loredo et al., 2018; Hobkirk et al., 2019). The other factor is a preference for risky large rewards (i.e., risk-taking) over secure small rewards (i.e., risk-aversion) in probability discounting (Green and Myerson, 2004). Two profiles of persons with drug use have been identified, one with highly impulsive decision-making and risk-taking, and the other with highly impulsive decision-making and risk-aversion (Green and Myerson, 2013; Nigg, 2017).

Defined as persons aged 10 to 19, adolescents are at a high level of risk for drug use involvement (World Health Organization, 2020). During this developmental stage, teenagers do not have a fully matured pre-frontal cortex, an area of the brain that is responsible for executive functioning skills, such as flexible thinking, self-control, and working memory (Bjork and Pardini, 2015; Moreira et al., 2015; van Duijvenvoorde et al., 2016; Nigg, 2017; Romer et al., 2017; Almy et al., 2018; Gibbons, 2019; McKewen et al., 2019; Meisel et al., 2019). The dual systems model posits that adolescent risk behavior is the result of an imbalance between high-reward systems and diminished cognitive control behavior (Mullan et al., 2011; Hanson et al., 2014; Willoughby et al., 2014; Janssen et al., 2015; Uroševic et al., 2015; Almy et al., 2018; Sherman et al., 2018; Meisel et al., 2019). Although useful, this model has faced criticism for lacking data that reflect risk taking in the real world. Additionally, for the discordance that arises between the peak of imbalance occurring between ages 14 and 16 and the peak of risky behavior occurring between ages 19 and 23 (chronic use, binge drinking, intoxication-related death) (Willoughby et al., 2014; Bjork and Pardini, 2015; Gibbons, 2019).

The elements proposed by the dual systems model (e.g., delay discounting and inhibition) have demonstrated the prediction of both onset use and frequency of use in persons without SRD, with odd ratios between 1.34 and 2.4, as well as explained variance between 9.1 and 43% (Fernie et al., 2013; Khurana et al., 2013; Hanson et al., 2014; Jonker et al., 2014; Day et al., 2015; Peeters et al., 2015, 2017; Uroševic et al., 2015; van Hemel-Ruiter et al., 2015; Richardson and Edalati, 2016). Although reward valuation and executive functioning are involved during the offset of risky behavior, it is important to identify other factors that contribute to the increase in consumption and consequential development of an SRD.

Therefore, the focus of this research will be on how to reduce factors that make people vulnerable to the development of an SRD rather than investigating how to eradicate risk-taking altogether. At a fundamental level, risk-taking behaviors are necessary for achieving new relationships, gaining independence, and coping with the daily challenges of life. To deal with these differences, it has been suggested to differentiate between adaptive and destructive risk-taking. Studies have reported that when using working memory as a mediator, delay discounting is higher in those with SRD and has been related to dependence severity; and that executive function is higher in those with less consumption (Khurana et al., 2015; Martínez-Loredo et al., 2018; Cassidy et al., 2020). A possible component that could improve the prediction of problematic drug involvement is antisocial traits (AT), characterized by callous-unemotional traits, inability to inhibit inappropriate behaviors and impulses, and a tendency toward deception and manipulation (Malesza and Ostaszewski, 2016). The inclusion of AT could be effective for prediction, as teenagers with drug use display more problem behaviors typical for those with AT (e.g., attention deficit hyperactivity disorder, oppositional defiant disorder, and conduct disorder) than those who do not use, and 77% of adolescents with SRD exhibit AT-related behaviors (Tucker et al., 2006; Hanson et al., 2014; Velásquez-Molina and Ordóñez-Huamán, 2015).

Additionally, studies have found that subjects with AT and SRD have steeper delay discounting than those with only SRD (Moreira et al., 2015; Moody et al., 2016). Even teenagers without SRD but with AT have more discount of delayed rewards than those without AT (Moreira et al., 2015; van Duijvenvoorde et al., 2016). Moreover, models that include AT have found them to be a significant predictor of drug involvement, and to have achieved better prediction accuracy than models that only include reward valuation and executive functions: odd ratios between 3.3 and 11.1, as well as the explained variance between 44 and 49% (Brook et al., 2012; Miranda et al., 2016; Squeglia et al., 2017). Furthermore, persons with AT and those with drug use tend to show cognitive inflexibility (CI) (Hanson et al., 2014; Broche-Pérez and Cortés-González, 2015; Hagen et al., 2016). CI refers to incapacity to inhibit a cognitive strategy or action sequence despite negative feedback (Flores et al., 2014). In consideration of individuals who maintain substance use despite the negative consequences related to SRD, it has been hypothesized that CI could intensify the reward valuation effect (Bjork and Pardini, 2015). Research studies that used a risk-taking behavioral task have reported that some participants displayed higher levels of risk-taking at the beginning of behavioral tasks, with an eventual shift to strategies that were less risky. Other participants continued to display higher levels of risk-taking for the entirety of the behavioral tasks (Xiao et al., 2013; Almy et al., 2018).

There are factors in addition to excessive reward valuation that are important when considering the onset of substance use. Researchers who studied substance use in relation to punishment valuation and stepper discounting (i.e., preference for large and delayed/insecure punishment instead of small and immediate/secure ones) have demonstrated their potential for prediction (Green and Myerson, 2013; Nigg, 2017). Likewise, increased punishment sensitivity and steeper delay discounting of losses have been related to alcohol and marijuana use (Jonker et al., 2014; Myerson et al., 2015; van Hemel-Ruiter et al., 2015; Mejía-Cruz et al., 2016).

Therefore, the main objective of this study is to identify differences between four types of drug involvement (non-use, experimental, moderate, and problematic) and five predictive factors (reward/gain, punishment/loss, discounting, CI, and AT). The second objective of this study is to evaluate how these variables contribute to predicting each type of involvement. This research goal is important given that only few studies have included these variables in the same study. This inclusion may facilitate the identification of interaction effects, resulting in predictions that are more accurate (Squeglia et al., 2017). Besides, identifying which variables are more relevant to each involvement type can be useful for improving the prevention of SRD development. In accordance with previously published research, we expect the moderate- and experimental-use groups to be predicted by high reward valuation (i.e., preference for small immediate and large risky rewards). In contrast, the proposed factors of antisocial traits, low punishment valuation (i.e., preference for large and delay/insecure losses), and cognitive inflexibility are expected to predict the problematic-use group.

## Method

### Participants

Fifty-two adolescents were selected for this study. The adolescents were middle or high school students from the south of Sonora, México. The inclusion criteria for participation were established by the World Health Organization (2020). Based on these criteria, the age range of 10 to 19 years was selected, and participants exhibiting any indication of psychotic or (hypo-)manic disorders were excluded. All the participants delivered an informed consent letter, which was signed by their parents or tutor. If a participant was 18 years or older, they signed the consent by themselves.

All the participants were living in the greater metropolitan area of the city at the time of the study. The protocol was approved by the Institutional Review Board of the Sonora Institute of Technology (ID 37). Additionally, all the participants provided the written informed consent following the Declaration of Helsinki, and they were not compensated with money for their participation.

### Measures

#### Inclusion Criteria Assessment

Mini International Neuropsychiatric Interview. A structured interview with dichotomy answers based on DSM-IV and ICD-10 was used. Each affirmative response was scored with one point. A score greater than three indicates a possible episode or disorder. The interview has test–retest reliability ≥ 0.75. The psychotic disorder section includes 10 questions, such as “Have you ever heard things other people couldn't hear, such as voices?” The (hypo-)manic episode section has nine questions such as “Are you currently feeling ‘up’ or ‘high’ or ‘hyper’ or full of energy?” (Sheehan and Lecrubier, 2006). Only these two sections were applied.

Montreal Cognitive Assessment (MoCA) for cognitive impairment detection. The MoCA assesses executive function, attention, abstraction, working memory, language, orientation, and visuospatial abilities. A score below 19 points indicates impairment. It has shown a Cronbach's α of 0.88 in Spanish-speaking Hispanics and 0.89 in the Mexican population, as well as convergent validity of 0.83 (Aguilar-Navarro et al., 2018).

Alcohol, Smoking, and Substance Involvement Screening Test (ASSIST). The ASSIST is a test developed for the WHO. It consists of eight questions about lifetime use, consumption, and consumption troubles related to the last 3 months. For example, “During the past 3 months, how often have you failed to do what was normally expected of you because of your use of (drug)?” The test includes questions about the use of alcohol, tobacco, cannabis, amphetamines, opioids, sedatives, cocaine, hallucinogens, and inhalants. Each substance is scored from questions two to seven. This test has shown an internal consistency of 0.8 and 0.87 (Tiburcio Sainz et al., 2016) in the Mexican population.

#### Discounting Tasks

Four different computer discounting tasks versions [Java^®^ (TM) Platform SE b version 7, for Windows^®^7 and 8] were applied: delay gains, delay losses, probability gains, and probability losses. Each of them consisted of a block of four practice trials and a block of 25 trials. The initial value of the large alternative was 3,000 Mexican pesos. The delays used were 1 week, 1 month, 6 months, 1 year, and 3 years. The probabilities were 10, 25, 50, 75, and 90%. All delay and probability discounting tasks used an adjusting-amount procedure that converges on the amount of an immediate certain outcome equal in subjective value to a delayed or probabilistic outcome (for a detailed description, see Du et al., 2002). These tasks have an internal consistency between 0.94 and 0.96 (Nguyen et al., 2018).

#### Cognitive Flexibility

Card Sorting Test (CST) includes 64 cards with figures. Each card varies in color (red, dark blue, blue, and brown), number of figures (one, two, three, and four), and shape (square, rhombus, trapezium, and octagon). A sheet with four cards is placed on top of a desk, and 64 cards are taken one by one. Each time, the participants must sort the card according to one of the three possible criteria and categorize it with one of the four cards presented on the desk. The evaluator indicates when an answer is correct or incorrect. The analysis units of both tests were perseverative errors and deferred perseveration. This test has a reliability of 0.8 in the Mexican population (Flores et al., 2014).

#### Antisocial Process Screening Device (APSD)

The APSD is a self-report scale of 20 items with a three-point Likert scale. The total score is obtained by adding points. The APSD includes questions about callous-unemotional (e.g., “feel bad when doing something wrong”), impulsivity (e.g., “acts without thinking”), and narcissism (e.g., “cons others to get what you want”). It has an internal consistency of 0.79 in Mexican adolescents (Mejía et al., 2019).

#### Procedure

There were two evaluation periods. The first period began on October 15, 2019 and finished on December 7, 2019. The second period began on April 20, 2020 and finished on May 4, 2020. During both periods, the University Linking Department made collaboration agreements and organized the selection of participating schools. For instruments and tests application, undergraduate psychology students were trained a week before the evaluation as part of their professional practice, social service, or volunteering.

A social worker was assigned to select the groups. Students belonging to selected groups were invited to participate in the research. Extra credit was offered to those who finished the final evaluation. Students who delivered a letter of signed informed consent were assessed in three sessions of 1 h, with a maximum of two sessions per week. A total of 12 students were excluded from the analysis, five students because of perpetual absence from school, one because of a significant MINI score on the (hypo-)manic episode evaluation, and six because of not answering the ASSIST.

#### Analysis

The participants were assigned to a group based on their ASSIST scores. The non-substance-use group was composed of participants who scored zero in all items of question one (never used any drug). The experimental substance-use group was composed of participants who scored three points or more in question one (tried drugs at least once in your life) but zero points in question two (no use in the last 3 months). The moderate substance-use group included participants who acquired low-risk scores, an alcohol score between 1 and 10, and other substances between 1 and 3. The problematic-use group consisted of participants who attained moderate risk scores, an alcohol score between 11 and 26, and other substances between 4 and 26.

Due to the coronavirus disease-2019 contingency, the assessments were suspended, and the researchers were unable to reach the sample size. Despite this setback, the obtained sample size met the minimum requirement for discriminant analysis: a minimum of five observations per variable and the group size being equal to or greater than the number of variables plus one (Hair et al., 2014). These requirements were achieved because the minimum number of observations for the seven employed variables was obtained (i.e., 35) and the smallest group size had more than eight participants; thus, the analysis continued.

Regarding CST, a single reaction response time faster than 200 milliseconds was eliminated. Concerning discounting tasks, indifference points were assessed with the algorithm of Johnson and Bickel (2008) to determine whether it was adequate to use parameter *k*. The suggested arrangements were employed. However, the number of participants for parameter *k* use was too low; hence area under the curve (AUC) was used (for details, see Myerson et al., 2001). To identify a non-random pattern in predictor variables, a missing data analysis was employed. Missing data randomness was determined by *t*-test and MCAR test [χ^2^(35) = 32.22, *p* = *0*.367]. Hence, mean group imputation was carried out for variables with <10% of missing data, and regression imputation for the group for variables with more than 10%. Normality criteria for small samples were employed: Shapiro–Wilks no significant, *z* skewness, and *z* kurtosis smaller than 1.96 as well as scores not exceeding 2.5 standard deviations (Hair et al., 2014). In the case of sociodemographic data, these criteria were also used for selecting a parametric or non-parametric test. In turn, predictor variables that did not gather criteria were transformed into a logarithmic or square root scale and re-tested. Transformations with better adjustments were kept. If criteria had not been gathered, the original scores were used, and all outliers were identified and normalized (Field et al., 2012). After that, distribution variables were re-tested, and adjustments with normality and equality of variances index were kept.

Discriminant function analysis with simultaneous estimation, varimax rotation, and cross-validation was carried out using SPSS version 23.0^®^ with syntax command. Additionally, the potency index was calculated to identify the discriminant contribution of each variable. Regarding function accuracy, the maximum chance criterion and proportional chance criterion for unequal groups was estimated, adding 25% to establish the classification threshold by chance. With the same purpose, the Q statistic of Press was obtained considering a critical value of 6.63 equivalent to *p* < *0*.01 (Hair et al., 2014).

Based on ANOVA included in discriminant function analysis, a *post hoc* with Gabriel test (for unequal groups) was made with variables that show means statistically different. The effect size was calculated considering ω^2^ values: small between 0.01 and 0.05, a medium between 0.06 and 0.13, and large ≥0.14 (Field et al., 2012). Additionally, statistical power (*P*) *post hoc* was estimated using G^*^Power, considering 0.8 an adequate power (Hair et al., 2014).

For diagnostic cases, correctly classified and misclassified cases were compared. First, the normality and equality of variances tests were made for determinate parametric or parametric tests with the same criteria already mentioned. After the effect size was calculated considering *r* values: ≥0.1 as small, ≥0.3 medium, and ≥0.5 large effect (Field et al., 2012).

## Results

### Group Characteristics

All the participants were unmarried, living with relatives, and within the 13–19 year age range (*M* = 15.54, *SD* = 1.64, females = 54.5%). The education year mean was 9.8 (*SD* = 1.7). On average, the adolescents with employment have worked 16.14 months (*SD* = 7.62). Furthermore, the education mean of the mother was 11.55 (*SD* = 2.4), and the education mean of the father was 11.83 (*SD* = 2.72). Additionally, there were no significant differences among the groups concerning sociodemographic characteristics, cognitive functioning, psychopathological symptoms, and family background (see Table 1).

**Table 1:** Sociodemographic characteristics per type of drug involvement.

**Variable**	**Non-use (*n =* 14)**		**Experimental use (*n =* 11)**		**Moderate- use (*n =* 17)**		**Problematic- Use (*n =* 10)**		**χ^2^ (3)**	***p***
	***n***	***%***	***n***	***%***	***n***	***%***	***n***	***%***		
**Gender**										
Female	8	57.1	5	45.5	9	52.9	6	60.0		
Male	6	42.9	6	54.5	8	47.1	4	40.0	*0*.53	0.912
Age*^a^*	15	4.0	16	5.0	17	6.0	15	4.0	4.41	0.221
MoCA*^a^*	25	6.0	26	11.0	26	12.0	28	8.0	3.74	0.291
Grade*^a^*	9	1.7	8	2.5	8.5	2.7	8.1	3.0	5.21	0.157
**HEL**										
Middle school	6	42.9	2	18.2	2	11.8	1	10.0		
High school	8	57.1	9	81.8	15	88.2	9	90.0	5.66	0.130
**Employment**										
No	13	92.9	9	81.8	14	82.4	9	90.0		
Yes	1	7.1	2	18.2	3	17.6	1	10.0	1.05	0.789
**PAYE**										
12 or less	9	69.2	7	63.6	11	64.7	8	80.0		
More of 12	4	30.8	4	36.4	6	35.3	2	20.0	0.85	0.837
**DAF**										
No	5	38.5	3	42.9	9	60.0	3	37.5		
Yes	8	61.5	4	57.1	6	40.0	5	62.5	1.73	0.629
**PIF**										
No	12	92.3	6	85.7	13	86.7	7	87.5		
Yes	1	7.7	1	14.3	2	13.3	1	12.5	*0*.29	0.961

*MoCA, Montreal Cognitive Assessment; HEL, highest educational level; PAYE, parents average years of education; DAF, drug abuse in the family; PIF, psychiatric illness in the family*.

a *Kruskal Wallis Test. Median, range, and H value are reported*.

### Discriminant Function Analysis

**Figure 1:**
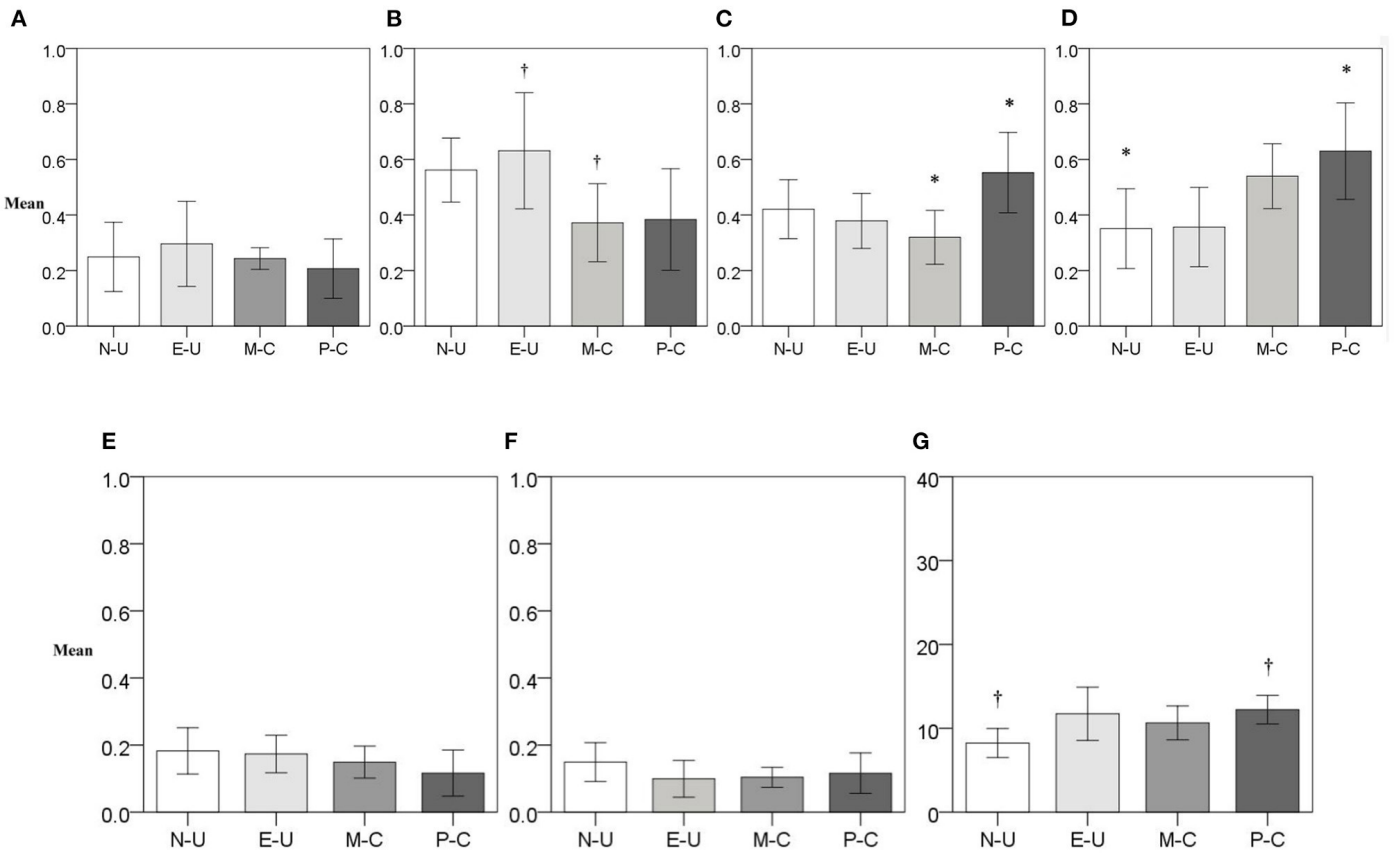
Predictor variables mean by type of drug involvement. **(A)** Delay Gain (Discounting Task), **(B)** Delay Loss (Discounting Task), **(C)** Probabilistic Gain (Discounting Task), **(D)** Probabilistic Loss (Discounting Task), **(E)** Perseverative Errors (CST), **(F)** Deferred Perseveration (CST), and **(G)** Antisocial Traits (APSD). N-U, non-use; E-U, experimental use; M-C, moderate-use; P-C, problematic use; CST, card sorting test; APSD, antisocial process screening device. ^†^*p* > *0.0*5. *p < *0.0*5.

After data transformation, only Probabilistic Gains show significant difference with normal distribution in the non-use group, and Delayed Gains had unequal variance. The equivalent covariance matrices (*Box's M* = 128.52, *p* = *0*.226) were enough to carry out a discriminant analysis. Moreover, the mean of four variables differs significantly between groups with medium to large effect size and low statistical power: probabilistic gains [*F*_(3,48)_ = 3.53, *p* = *0*.022, ω^2^ = 0.13, *P* = *0*.05], delayed losses [*F*_(3,48)_ = 3.11, *p* = *0*.035, ω^2^ = 0.11, *P* = 06], probabilistic losses [*F*_(3,48)_ = 4.15, *p* = *0*.011, ω^2^ = 0.15, *P* = *0*.04], and antisocial traits [*F*_(3,48)_ = 2.96, *p* = *0*.041, ω^2^ = 0.21, *P* = *0*.18]. Although *post hoc* only confirmed differences in probabilistic gains (problematic use vs. moderate use: *p* = *0*.013, 95% *CI* = 0.43–0.04) and probabilistic losses (problematic use vs. non-use: *p* = *0*.033, 95% *CI* = 0.02–0.54). In delayed losses, the closest comparison to significance was between moderate use and experimental use (*p* = *0*.074, 95% *CI* = −0.02 to 0.53), whereas antisocial traits were associated with problematic use (*p* = *0*.063, 95% *CI* = −8.1 to 0.13) (see Figure 1).

Three functions result from discriminant analysis, together explaining 60.1% of the dependent variable variance. In the first function, delayed losses and probabilistic losses contributed the most, whereas, in the second function, antisocial traits and deferred perseveration contributed the most. Moreover, perseverative errors were very close to 0.4 in the second function. However, its value on the first two functions was very similar; thus, it is not clear to which it belongs. In the third function, only probabilistic gains had a substantial contribution to prediction. Regarding general contribution, probabilistic losses and delayed losses had the highest potency index. These were followed by probabilistic gains, antisocial traits, deferred perseveration, and perseverative errors, which had similar inputs. As for delayed gains, it had a minimal contribution as individually as overall with absolute values.

Concerning prediction accuracy, all hit ratios (overall and group percentage of correctly classified cases) were above the classification thresholds. Likewise, the Q value of Press was higher than the critical value (6.63) (see Table 2). Therefore, this model was better than random classification at a level of *p* < *0*.01, with non-use and problematic use groups being the most correctly classified (see Table 3). Additionally, function one distinguished among non-use, experimental, moderate, and problematic use; whereas function two differentiated between non-use and experimental-use. Nonetheless, the moderate and problematic consumer cases overlapped, and their centroids were very close. Lastly, moderate-use cases were the most dispersed.

**Table 2:** Functions in simultaneous discriminant function analysis.

**Function test**	**Wilks's λ**	**χ^**2**^**	***df***	***p***	**Percentage of explained variance**
1 at 3	0.404	41.25	21	0.005	40.7
2 at 3	0.681	17.49	12	0.132	10.7
3	0.831	8.42	5	0.135	8.7

**Table 3:** Standardized discriminant functions coefficients (rotated function structure matrix) and potency index.

**Predictor variable**	**Function**			**Potency Index**
	**1**	**2**	**3**	
Delay Loss (Discounting Task)	**0.736**	0.214	0.150	0.348
Probabilistic Loss (Discounting Task)	**-0.761**	0.170	0.070	0.364
Antisocial Traits (APSD)	−0.007	**0.833**	0.117	0.141
Deferred Perseveration (Card Sorting Test)	−0.098	**-0.767**	0.089	0.124
Perseverative Errors (Card Sorting Test)	0.358	0.398	−0.161	0.115
Probabilistic Gain (Discounting Task)	−0.072	0.067	**0.952**	0.170
Delay Gain (Discounting Task)	0.047	−0.075	0.040	0.003

*APSD, antisocial process screening device. Discriminant loadings above 0.400 are in bold*.

#### Cross-Validation

Prediction accuracy decreased in all levels, except in the moderate-use group where the percentage was the same. Nonetheless, the hit ratios were kept above analysis estimation thresholds, except for the problematic-use group located below the maximum chance criterion by 0.9%. Moreover, the Q value of Press also kept higher than the critical value (see Table 4). Hence, despite decrements in classification accuracy, this model was still better than random classification at the level of *p* < *0*.01.

**Table 4:** Classification analysis for type of drug involvement.

**Actual group**	***n***	**Predicted group membership a Kruskal**							
		**Non-use**		**Experimental use**		**Moderate-use**		**Problematic-use**	
		***n***	***%***	***n***	***%***	***n***	***%***	***n***	***%***
**Estimation*^a^***									
Non-use	14	11	78.6	0	0.0	0	0.0	3	21.4
Experimental use	11	2	18.2	7	63.6	2	18.2	0	0.0
Moderate-use	17	4	23.5	1	5.9	10	58.8	2	11.8
Problematic-use	10	1	10.0	0	0.0	2	20.0	7	70.0
**Cross-validation*^b^***									
Non-use	14	7	50.0	4	28.6	0	0.0	3	21.4
Experimental use	11	2	18.2	5	45.5	3	27.3	1	9.1
Moderate-use	17	4	23.5	1	5.9	10	58.8	2	11.8
Problematic-use	10	1	10.0	1	10.0	4	40.0	4	40.0

a *Overall percentage of correctly classified cases = 67.3%*.

b *Overall percentage of correctly classified cases = 50%*.

In the non-use group, three cases were misclassified, from which only two had a higher probability of being classified in the wrong group in contrast with the likelihood of the correct one. In the experimental use group, all the individuals had a higher probability of being misclassified. In the moderate and problematic consumer groups, the cases had almost a double or more than double likelihood of being assigned to the wrong group. Furthermore, in the moderate-use group, most of the participants were classified as non-use. Thus, two analyses were carried out. The first analysis grouped all the misclassified cases together. The second analysis examined the moderate-use groups, differentiating between those classified as non-use and those with other classification.

In the non-use group, misclassified cases showed larger AUC in probabilistic gains (*U* = 0, *p* = *0*.01, *r* = −0.18) and smaller AUC in delayed losses (*U* = 3, *p* = *0*.036, *r* = −0.15) than correctly classified, both with a small effect size. In the experimental-use group, misclassified cases had less antisocial traits than correctly classified cases, with a large effect size [*t*(9) = −3.03*, p* = *0*.014, *r* = 0.71]. In the moderate-use group, misclassified cases showed larger AUC in probabilistic gains [*t*(15) = 3.53, *p* = *0*.003, *r* = 0.67] and in delayed losses (*U* = 6, *p* = *0*.005, *r* = −0.17), as well as more deferred perseveration [*t*(15) = 2.36, *p* = *0*.032, *r* = 0.52]. Lastly, in the problematic-use group, significant differences were not found.

The analysis with only moderate substance use distinguished those misclassified values as non-use (misclassified 1) and others (misclassified 2). There were significant differences among correctly classified and two misclassified groups in probabilistic gains [*H*(2) = 7.05, *p* = *0*.017], delayed losses [*H*(2) = 8.49, *p* = *0*.005], and antisocial traits [*H*(2) = 11.05, *p* = 0]. *Post hoc* with Bonferroni correction (critical value *p* = *0*.017) revealed that misclassified 2 had larger AUC in probabilistic gains than correctly classified with small effect size (*U* = 1, *p* = *0*.014, *r* = −0.18). In turn, misclassified 1 showed lower antisocial traits than correctly classified with small effect size (*U* = 0, *p* = *0*.001, *r* = −0.2); it was also smaller than misclassified 2, albeit it does not reach enough significance (*U* = 0, *p* = *0*.029, *r* = −0.81). Regarding delayed losses, correctly classified had smaller AUC than misclassified 1 (*U* = 4, *p* = *0*.024, *r* = −0.16) and misclassified 2 (*U* = 2, *p* = *0*.028, *r* = −0.17); however, these comparisons did not reach required significance.

## Discussion

This study aimed to identify the predictive power of gain and loss discounting, cognitive inflexibility, and antisocial traits in four types of drug use. The characteristics of the sample used were adequate. All the groups were equal in terms of sociodemographic data, global cognitive functioning, psychopathological symptoms, and family background. In comparison to other research studying adolescent drug use, the participants presented comparable drug use trends (primarily alcohol, tobacco, and marijuana) (Brook et al., 2012; Janssen et al., 2015; Miranda et al., 2016; Richardson and Edalati, 2016; Tomczyk et al., 2016; Peeters et al., 2017; Martínez-Loredo et al., 2018; Mendoza Armenta et al., 2020).

Regarding the results of ANOVA derived from discriminant analysis, the non-use group had lower AT than the problematic-consumption group. However, the non-use group did not have the lowest levels as the other groups had no significant differences. Additionally, the outcome for Probabilistic Losses was contrary to what had been predicted. Probabilistic losses discounted more punishments (small AUC: preferred large and insecure losses) than the problematic consumer group. The first finding supports other studies that have also found that groups with heavy and chronic substance use show more antisocial behavior than the non-substance use group (Brook et al., 2012; Hanson et al., 2014; Squeglia et al., 2017). Brook et al. (2012) found that teenagers with chronic drug use had higher depression, anxiety, and interpersonal hypersensitivity than those without use. Since the first depression and anxiety are related to high punishment sensitivity (Carver and White, 1994; Ernst et al., 2006), this could be related to how the non-use group in this study showed more discounting of losses (less sensitivity to unlikely punishments) than the problematic consumer group. This finding indicated that adolescents without drug use are characterized by low AT and risky decisions, whereas teenagers with problematic consumption have more AT and risk aversion toward losses.

The moderate-use group showed differences from the experimental-use group in an increased display of discounting of delayed losses (small AUC) and presented less risk-taking than the problematic-use group (small AUC in probabilistic gains). As for delayed losses, research in adults has found similar results. Those with higher alcohol and marijuana consumption exhibit higher discounting of delayed losses than those without use (Myerson et al., 2015; Mejía-Cruz et al., 2016). This tendency indicates that those with moderate consumption levels are less sensitive to delayed negative consequences. The research on risk-taking among adolescents is not conclusive. Some studies have not found differences between heavy and moderate substance use (Mullan et al., 2011). Other studies have found high risk-taking in moderate-use during adolescence (Brook et al., 2012). This suggests that with moderate use, the two profiles suggested by Green and Myerson (2013), high risk-taking and risk inversion, are present. The non-use and experimental use groups were more sensitive to negative consequences in the long term (large AUC in delayed losses) than the other groups. However, these two groups also underestimated unlikely negative outcomes (small AUC in probabilistic losses). A negative relationship between the delayed losses and probabilistic losses has also been observed in other studies (Green and Myerson, 2013).

When assessing predictive power, the frequency of alcohol showed a negative relationship, although it did not reach statistical significance. This result is similar to research that does not distinguish between delay and probability as factors (van Hemel-Ruiter et al., 2015). Another study found that executive functioning was a significant predictor when used as a mediator (Jonker et al., 2014). Perhaps this explains how the perseverative errors (an indicator of cognitive inflexibility) almost reached a significant discriminant loading. These findings suggest the importance of using both dimensions of punishment valuation, which each presented contrary to the hypothesis, as well as to include executive functioning.

It was not expected that only one function would make a distinction between non-use and experimental use. Here, high antisocial traits and low cognitive inflexibility were useful to discriminate experimental use from non-use. This corresponds with several studies in which antisocial traits have shown to be a good predictor of drug use trajectory (Brook et al., 2012; Miranda et al., 2016; Tomczyk et al., 2016). We did not find differences among the groups in cognitive inflexibility, and this finding is contradictory to other studies (Mullan et al., 2011; Squeglia et al., 2017). Thereby, in consideration of laws that enforce the legal drinking age and non-prescription substance use, adolescent drug experimentation requires one to break the law. Hence, experimental use could be characterized by having more antisocial traits than non-use. It is important to note that the AT among experimental users is not at the pathological level of those with SRD. Likewise, Tucker et al. (2006) found that experimental users showed more antisocial behavior than abstainers, but less antisocial behavior than frequent users. Moreover, experimental users tend to display more sensation-seeking behavior than non-users (Khurana et al., 2015).

This finding is particularly noteworthy because most studies have allocated non-users and experimental users into the same group. These results suggest that the two groups have two distinct profiles (Brook et al., 2012; Khurana et al., 2015; Tomczyk et al., 2016). Although this function did not reach sufficient statistical significance, discriminant loadings were high; the non-use group had the best classification accuracy for estimation analysis and the second best for cross-validation.

It was expected that loss discounting, cognitive inflexibility, and antisocial traits would predict problematic consumption. However, when those factors interacted with high risk-taking (large AUC in probabilistic gains) in the third function, the analysis did not show accurate discrimination among the groups. This finding supports similar studies where risk-taking was not found to be a significant predictor of substance use (Janssen et al., 2015), and in having similar percentages of explained variance (9.1%) (Hanson et al., 2014).

In this study, delayed gains did not contribute to explaining the lowest discriminant loadings and potency index. This finding is similar to cross-sectional research with an age range of 12–18 years, where delayed gains were not a predicting factor (Janssen et al., 2015). Alternatively, research involving older teenagers (15–19 years) found delayed gains to be an important predicting factor (Richardson and Edalati, 2016; Cassidy et al., 2020). In fact, cohort studies on adolescents have found that delayed gains are mediators to drug use (Khurana et al., 2013, 2015). This could be related to an age effect as impulsive decision-making demonstrates a linear decrease from childhood to adulthood (Moreira et al., 2015; Nigg, 2017; Romer et al., 2017; McKewen et al., 2019). This also suggests that differences in delayed gains are not large in early adolescence, but that they are larger during late adolescence and adulthood (Green and Myerson, 2013; Mejía Cruz et al., 2015; Mejía-Cruz et al., 2016; Moreira et al., 2015; Myerson et al., 2015; Moody et al., 2016; Quisenberry et al., 2016; Richardson and Edalati, 2016; Hobkirk et al., 2019).

Considering the results from the diagnostic cases, all misclassifications into the non-use group were identified as problematic consumption, which presented significantly higher risk-taking (large AUC in probabilistic gains) and delay discounting of negative outcomes (small AUC in delayed losses), indicating these adolescents may display their riskiest drug involvement at a future time point or during adulthood (Willoughby et al., 2014; Bjork and Pardini, 2015; Gibbons, 2019).

Regarding the experimental-use group, misclassified cases did not show a particular tendency as did the previous group. Some of the participants were classified as non-use and the others as moderate-use. This could suggest that the measurement of antisocial traits did not accurately discriminate. Perhaps making changes to the current measure or adding a conduct disorder questionnaire could improve the model or the second function. Such adaptations may better facilitate the research as other studies have demonstrated these measurements to be good indicators of antisocial traits (Brook et al., 2012; Tomczyk et al., 2016; Squeglia et al., 2017).

The findings from the three moderate use cases coincided with another study that identified three distinct moderate drug use trajectories in adolescence. The first trajectory (continuing infrequent consumption in adulthood) showed higher rates of depression (depression symptomology or indication of major depressive episode) but low antisocial behavior, whereas the misclassified case had a large AUC in delayed losses and low AT. The second trajectory (with an increase of tobacco use into adulthood) was marked with high rates of antisocial behavior, as well as low risk-taking and depression (same as above), likewise correctly classified: high APSD score, aside from small AUC in probabilistic gains and delayed losses. The third trajectory (with an increase in marijuana use in adulthood) was marked with high risk-taking behavior and depression (Brook et al., 2012). As previously mentioned, this phenomenon could be related to the relationship between high punishment sensitivity and depression (Carver and White, 1994; Ernst et al., 2006).

One strength of this study was that the researchers recruited adolescents without substance related disorders. This was done in order to avoid any drug-related effects on executive functioning (cognitive flexibility) due to damage from chronic and heavy substance consumption (Bjork and Pardini, 2015). Moreover, this proposal achieved higher explained variance than other studies that used the same variables separately, although it is important to note that the low statistical power in ANOVA highlights the importance of replicating this study with a larger sample size. Additionally, it is necessary to use a holdout sample for validation, which would thus discard some bias in terms of external validation (Hair et al., 2014).

In conclusion, these findings suggest that discounting of delayed losses plays a relevant role in the frequent use of substances (moderate- and problematic-use). Hence, it is necessary to look for interventions that achieve a high level of awareness among adolescents regarding drug-related damages, such as SRD, and potential negative consequences. It is important not to underestimate the magnitude of a potential consequence related to drug use, especially in consideration of the high-risk probabilities demonstrated by the different drug use trajectories. Further, these findings demonstrated the relevance of antisocial traits, even for experimental consumption. Therefore, an intervention that diminishes antisocial traits could prevent adolescents from taking drugs. It is important for such intervention programming to consider the different drug use trajectories and how maturation-related changes can account for the discounting of delayed gains. Therefore, it would be further necessary to identify specific factors that distinguish moderate substance use from problematic substance use and to consider additional variables, such as depression and anxiety. These efforts could help to precisely identify which variables are active during the development of SRD. That said, it is important to note that this study is limited in that the researchers gathered a large sample size and yet received a low statistical power. It is suggested that the study be replicated with a large sample.

## Data Availability Statement

The raw data supporting the conclusions of this article will be made available by the authors, without undue reservation.

## Ethics Statement

The studies involving human participants were reviewed and approved by the Institutional Review Board of the Sonora Institute of Technology (ID 37). Written informed consent to participate in this study was provided by the participants'alegal guardian/next of kin.

## Author Contributions

LH and DM designed the study and wrote the protocol. LH conducted literature searches and provided summaries of previous research studies. DM wrote the first draft of the manuscript. LA-C conducted the statistical analysis. All authors contributed to and have approved the final manuscript.

## Conflict of Interest

The authors declare that the research was conducted in the absence of any commercial or financial relationships that could be construed as a potential conflict of interest.
