# Supplementary material for: Discounting, Cognitive Inflexibility, and Antisocial Traits as Predictors of Adolescent Drug Involvement
Source: Front Psychol. 2021 Jun 17;12:676250. doi: 10.3389/fpsyg.2021.676250 (PMC8245670; doi:10.3389/fpsyg.2021.676250)
Supplement: Supplementary file 1 [file Table_1.DOCX]

**Complementary Material**

*Substances Used by Group at Least Once in Life*

| Substance | Experimental use | | Moderate-use | | Problematic-use | |
| --- | --- | --- | --- | --- | --- | --- |
|  | (*n*=11) | | (*n*=17) | | (*n*=10) | |
|  | *n %* | | *n %* | | *n %* | |
| Tobacco | 2 | 18*.*2 | 4 | 23*.*5 | 7 | 70*.*0 |
| Alcohol | 10 | 90*.*9 | 17 | 100*.*0 | 10 | 100*.*0 |
| Marijuana | 0 | 0*.*0 | 1 | 5*.*9 | 4 | 40*.*0 |
| Sedatives | 0 | 0*.*0 | 0 | 0*.*0 | 4 | 40*.*0 |
| Cocaine | 0 | 0*.*0 | 0 | 0*.*0 | 1 | 10*.*0 |
| Amphetamine | 0 | 0*.*0 | 0 | 0*.*0 | 0 | 0*.*0 |
| Inhalants | 0 | 0*.*0 | 0 | 0*.*0 | 0 | 0*.*0 |
| Hallucinogens | 0 | 0*.*0 | 0 | 0*.*0 | 0 | 0*.*0 |
| Opioids | 0 | 0*.*0 | 0 | 0*.*0 | 0 | 0*.*0 |
| Other | 0 | 0*.*0 | 0 | 0*.*0 | 0 | 0*.*0 |

*Substances Used by Group in the Past Three Months*

Substance Moderate-use Problematic-use

(*n*=17) (*n*=10)

*n % n %*

| Tobacco | 2 | 11*.*8 | 7 | 70*.*0 |
| --- | --- | --- | --- | --- |
| Alcohol | 16 | 94*.*1 | 10 | 100*.*0 |
| Marijuana | 1 | 5*.*9 | 3 | 30*.*0 |
| Sedatives | 0 | 0*.*0 | 2 | 20*.*0 |
| Cocaine | 0 | 0*.*0 | 0 | 0*.*0 |
